# RAD51-Mediated DNA Homologous Recombination Is Independent of *PTEN* Mutational Status

**DOI:** 10.3390/cancers12113178

**Published:** 2020-10-29

**Authors:** Asha Sinha, Ali Saleh, Raelene Endersby, Shek H. Yuan, Chirayu R. Chokshi, Kevin R. Brown, Bozena Kuzio, Tiina Kauppinen, Sheila K. Singh, Suzanne J. Baker, Peter J. McKinnon, Sachin Katyal

**Affiliations:** 1 Department of Pharmacology and Therapeutics, University of Manitoba, Winnipeg, MB R3E 0T6, Canada; sinhaa@myumanitoba.ca (A.S.); asaleh2@cancercare.mb.ca (A.S.); hyuan@cancercare.mb.ca (S.H.Y.); bkuzio@cancercare.mb.ca (B.K.); Tiina.Kauppinen@umanitoba.ca (T.K.); 2 Research Institute in Oncology and Hematology, CancerCare Manitoba, Winnipeg, MB R3E 0V9, Canada; 3 Telethon Kids Institute, Perth Children’s Hospital, 15 Hospital Avenue, Perth, WA 6009, Australia; Raelene.Endersby@telethonkids.org.au; 4 Centre for Child Health Research, University of Western Australia, 15 Hospital Avenue, Perth, WA 6009, Australia; 5 Department of Biochemistry and Biomedical Sciences, McMaster University, 1200 Main Street West, Hamilton, ON L8S 4L8, Canada; chokshc@mcmaster.ca (C.R.C.); ssingh@mcmaster.ca (S.K.S.); 6 Donnelly Centre, University of Toronto, 160 College St, Toronto, ON M5S 3E1, Canada; bioboy99@gmail.com; 7 Neuroscience Research Program, Kleysen Institute for Advanced Medicine, Health Sciences Centre, 710 William Avenue, Winnipeg, MB R3E 0Z3, Canada; 8 Department of Surgery, Faculty of Health Sciences, McMaster University, 1200 Main Street West, Hamilton, ON L8S 4L8, Canada; 9 Department of Developmental Neurobiology, St Jude Children’s Research Hospital, Memphis, TN 38105-3678, USA; Suzanne.Baker@STJUDE.ORG; 10 Department of Genetics, St Jude Children’s Research Hospital, Memphis, TN 38105-3678, USA; Peter.McKinnon@STJUDE.ORG

**Keywords:** PTEN, RAD51, homologous recombination, DNA damage, γH2AX foci, RAD51 foci, HRD, alkaline comet assay, PARP inhibitor, olaparib, base excision repair, synthetic lethality, combination therapy, glioblastoma multiforme, brain tumor initiating cells, RNA expression

## Abstract

**Simple Summary:**

PTEN is an important tumor suppressor that is frequently mutated in malignancy. *PTEN* mutational loss has been associated with reduced RAD51 expression and homologous recombination deficiency (HRD), however; recent studies have failed to recapitulate these findings. Here, we show that RAD51 expression, foci formation and homologous recombination repair activity are unaltered in normal and tumorigenic PTEN-deficient cells and patient samples. Furthermore, we show that PTEN-deficient tumor cell lines do not synergize with the clinical PARP inhibitor olaparib, underscoring a need to discontinue its use in treating patients with PTEN-deficient tumors that do not otherwise exhibit HRD.

**Abstract:**

PTEN mutation occurs in a variety of aggressive cancers and is associated with poor patient outcomes. Recent studies have linked mutational loss of *PTEN* to reduced RAD51 expression and function, a key factor involved in the homologous recombination (HR) pathway. However, these studies remain controversial, as they fail to establish a definitive causal link to RAD51 expression that is PTEN-dependent, while other studies have not been able to recapitulate the relationship between the PTEN expression and the RAD51/HR function. Resolution of this apparent conundrum is essential due to the clinically-significant implication that PTEN-deficient tumors may be sensitive to poly (ADP-ribose) polymerase (PARP) inhibitors (PARPi) commonly used in the clinical management of *BRCA*-mutated and other HR-deficient (HRD) tumors. Methods: Primary *Pten*-deficient (and corresponding wild-type) mouse embryonic fibroblasts (MEFs) and astrocytes and *PTEN*-null human tumor cell lines and primary cells were assessed for RAD51 expression (via the Western blot analysis) and DNA damage repair analyses (via alkali comet and γH2AX foci assays). RAD51 foci analysis was used to measure HR-dependent DNA repair. *Xrcc2*-deficient MEFs served as an HR-deficient control, while the stable knockdown of *RAD51* (*shRAD51*) served to control for the relative RAD51/HR-mediated repair and the phospho-53BP1 foci analysis served to confirm and measure non-homologous end joining (NHEJ) activity in PTEN-deficient and *shRAD51*-expressing (HRD) lines. Cell proliferation studies were used to measure any potential added sensitivity of *PTEN*-null cells to the clinically-relevant PARPi, olaparib. RAD51 levels and DNA damage response signaling were assessed in PTEN-mutant brain tumor initiating cells (BTICs) derived from primary and recurrent glioblastoma multiforme (GBM) patients, while expression of *RAD51* and its paralogs were examined as a function of the *PTEN* status in the RNA expression datasets isolated from primary GBM tumor specimens and BTICs. Results: *Pten* knockout primary murine cells display unaltered RAD51 expression, endogenous and DNA strand break-induced RAD51 foci and robust DNA repair activity. Defective HR was only observed in the cells lacking *Xrcc2*. Likewise, human glioblastoma multiforme (GBM) cell lines with known PTEN deficiency (U87, *PTEN*-mutated; U251 and U373, *PTEN*-null) show apparent expression of RAD51 and display efficient DNA repair activity. Only GBM cells stably expressing shRNAs against *RAD51* (*shRAD51*) display dysfunctional DNA repair activity and reduced proliferative capacity, which is exacerbated by PARPi treatment. Furthermore, GBM patient-derived BTICs displayed robust RAD51 expression and intact DNA damage response signaling in spite of *PTEN*-inactivating mutations. RNA expression analysis of primary GBM tissue specimens and BTICs demonstrate stable levels of *RAD51* and its paralogs (*RAD51B, RAD51C, RAD51D, XRCC2, XRCC3*, and *DMC1*), regardless of the *PTEN* mutational status. Conclusions: Our findings demonstrate definitively that PTEN loss does not alter the RAD51 expression, its paralogs, or the HR activity. Furthermore, deficiency in PTEN alone is not sufficient to impart enhanced sensitivity to PARPi associated with HRD. This study is the first to unequivocally demonstrate that PTEN deficiency is not linked to the RAD51 expression or the HR activity amongst primary neural and non-neural *Pten*-null cells, PTEN-deficient tumor cell lines, and primary *PTEN*-mutant GBM patient-derived tissue specimens and BTICs.

## 1. Introduction

*PTEN* encodes a phosphatidylinositol-3,4,5-trisphosphate 3-phosphatase containing a tensin-like domain and a catalytic domain typical of those of the dual-specificity protein tyrosine phosphatases [1,2]. Unlike most protein tyrosine phosphatases, PTEN preferentially dephosphorylates phosphoinositide substrates. PTEN is involved in diverse biological processes, functioning as an important negative regulator of the phosphatidylinositol-3,4,5-trisphosphate kinase-signaling cascade in several downstream cellular processes, including cell regulation and apoptosis. Functioning as a tumor suppressor, PTEN is one of the most commonly-mutated (inactivated) genes in human cancer, including glioblastoma multiforme (GBM), breast, prostate, endometrium, ovary, colon cancers, melanoma, and lymphoma [3,4]. Tumors featuring *PTEN* mutations are characterized by pronounced genomic instability and chromosomal defects [5].

Homologous recombination (HR) is a critical ATP-dependent DNA double-strand break repair (DSBR) pathway, particularly active in the G1-S phase of the cell cycle [6,7,8,9], wherein a template strand invades base-paired strands of homologous DNA molecules to guide repair of damaged DNA bases [10]. The RAD51 recombinase protein forms a tripartite complex with XRCC2 and BRCA2 that associates with additional co-factors and RAD51 paralog complexes to mediate this form of homologous sister-chromatid guided DSBR. Pathogenic germline and acquired somatic mutation, promoter hypermethylation, or other as yet to be identified mechanisms [11,12,13,14] can result in a complex loss of function leading to HR deficiency (HRD). HRD reduces overall DNA repair fidelity thus impacting cellular survival amongst dividing cell populations and can promote early tumorigenic events implicated in a number of malignancies, including breast, ovarian, brain, and endometrial cancers. Cellular survival in the context of HR inactivation is thought to be mediated via a combination of classical non-homologous end joining (c-NHEJ)-mediated DSBR, alternative end joining (alt-EJ), and/or base excision repair (BER) [15]. Nevertheless, tumors featuring bi-allelic HR-associated mutations offer a unique therapeutic opportunity that has heralded the use of inhibitors that target poly (ADP-ribose) polymerase (PARP) [16,17,18], a critical enzyme that functions as a DNA strand break sensor and activator of the BER response and other end joining repair pathways [19,20,21]. This “synthetic lethal” therapeutic strategy successfully exploits the tumor’s HRD and accompanying proliferation/replication stress whereby PARP inhibition/inhibitors (PARPi) target the cell’s outstanding break repair capacity. This approach has been successful in the treatment of breast, ovarian, and endometrial cancer patients that feature bi-allelic *BRCA1/2* mutations/deletion [17,22,23,24] and recently expanded clinical usage in lung, prostate, pancreatic, colon cancer and other cancer patients featuring a variety of tumors featuring HRD [18,25,26,27,28].

A number of studies have implicated PTEN in the cellular expression of RAD51 (and/or its paralogs), linking PTEN to cellular HR and DSBR [29,30,31]. Likewise, studies have attributed tumors with PTEN-deficiency as having enhanced sensitivity to PARP inhibition, purportedly due to PTEN-associated loss of RAD51 function and HRD, similar to *BRCA* loss [32,33]. However, these findings have been met with controversy, as more recent studies have shown opposite conclusions: that PTEN-deficient cells do not display nor attenuated RAD51 expression nor HRD [34,35,36,37]. The continued uncertainty surrounding the PTEN-RAD51 connection adversely affects scientific research, forums, and academia as differing viewpoints yield conflicting inferences, assumptions and factor increasingly prominently in translational research, drug development, and clinical care.

To delineate any potential role for PTEN in the regulation of the RAD51/HR-dependent activity, we used a systematic approach to evaluate RAD51 expression, DNA repair activity, and PARPi responses in diverse PTEN-deficient primary and cancer cell models. Here, we demonstrate that PTEN-deficient cells retain RAD51 expression and activity, while HR and DNA repair activity in general remain robust independent of the *PTEN* status. Consequently, we show that *PTEN*-null/mutant cells show no overt HR-dependent sensitivity to PARP1 inhibition, indicating that any putative PARP-dependent tumor ablation in *PTEN*-null cells likely involves alternative mechanisms. These studies demonstrate unequivocally that PTEN does not impact RAD51 expression or affect cellular HR/DNA damage repair activity.

## 2. Materials and Methods

### 2.1. Generation of Primary Cells from Pten-Deficient Mice

Mouse experiments were approved by the Institutional Animal Care and Use Committee (protocol number 278). Primary astrocytes were prepared [38] from cortices of postnatal day 2 (P2) brains derived from controls (*Pten^+/+^* and *Pten^LoxP/+^*) and conditional *Pten* (*Pten^LoxP/LoxP^*) [39]. Cortices were dissociated by passage through a 5 mL pipette and cells were resuspended in Dulbecco’s modified Eagle medium and Ham’s nutrient mixture F-12 (1:1 DMEM/F12, Gibco-BRL) supplemented with 10% fetal bovine serum (*v/v*), 1x glutamax, 100 U/mL penicillin, 100 mg/mL streptomycin, and 20 ng/mL epidermal growth factor (EGF; Millipore). Primary astrocytes were established in Primeria T-25 tissue culture flasks (Falcon) at 37 °C in a humidified CO_2_-regulated (5%) incubator. Following cell establishment, astrocytes at 25% confluency were transduced with retroviral Cre recombinase (MSCV-creGFP). DNA from green fluorescent protein (GFP)-positive cells was isolated and underwent PCR analysis to validate efficient Cre-mediated deletion of floxed sites within the *Pten* locus [39,40]. Immunoblotting analysis using a rabbit anti-PTEN monoclonal antibody (9188, Cell Signaling Technologies) was also used to confirm concordant PTEN expression status.

Primary mouse embryonic fibroblasts (MEFs) were prepared from an E13.5 embryonic mesenchyme [41]. Tissue was minced using dissection scissors, trypsinized, and resuspended in a Dulbecco’s modified Eagle medium (DMEM) supplemented with 10% fetal bovine serum (*v/v*), 1x glutamax, 100 U/mL penicillin and 100 μg/mL streptomycin (1× Pen/Strep), and β-mercaptoethanol and established in T-25 tissue culture flasks (Falcon) at 37 °C in a humidified CO_2_-regulated (5%) incubator. Similar to the astrocyte preparation, deletion of *Pten* within primary MEFs occurred via expression of retroviral *MSCV-creGFP*, followed by PCR validation and Western analysis as outlined above.

### 2.2. PTEN-Deficient Cancer Cell Lines and RNA Interference

*PTEN*-mutant (U87MG) and *PTEN*-deleted (U251MG and U373MG) GBM cell lines were originally obtained from American Type Culture Collection (ATCC) (Manassas, VA, USA) and maintained in a DMEM supplemented with 5% fetal bovine serum (*v/v*), 1× GlutaMAX, 100 U/mL penicillin, 100 μg/mL streptomycin. The Burkitt lymphoma cell line, BJAB, was grown in RPMI 1640 supplemented with 1× Pen/Strep and 1× GlutaMAX and 10% fetal bovine serum. The use of all cell lines was restricted to passages (P) 5 to P25. Lentiviral particles were generated from MISSION^®^ shRNA plasmid DNA constructs (Millipore-SIGMA, St. Louis, MO, USA) to deliver and stably express shRNAs in GBM cell lines to diminish human RAD51 expression (Figure S1). Lentivirus was made via co-transfection of pLKO.1-puro plasmids with MISSION^®^ lentiviral packaging DNA (Millipore-Sigma, SHP001) into sub-confluent 293LTV cells (Cell Biolabs Inc, San Diego, CA, USA) using XtremeGene HD (Millipore-SIGMA). Lentivirus was collected 48–96 h later and filtered (0.45µm pore size). GBM cell lines were transduced with lentiviral particles for 48 h at 37 °C followed by puromycin (1 μM) selection for 72 h. Western analysis was used to confirm shRNA-mediated knockdown of RAD51 expression. Following transduction of 3 independent *shRAD51* constructs into GBM cells and the subsequent Western analysis, TRCN0000329686 was selected for further knockdown studies due to its highest reduction in RAD51 protein expression (Figure S1). Non-target scrambled (*SCM*) shRNA and lentiviral TurboGFP (Millipore-SIGMA) expression cDNAs were used as negative and positive transduction controls, respectively.

### 2.3. Isolation and Propagation of GBM Patient Brain Tumor Initiating Cells (BTICs)

Human GBM samples from primary (treatment-naïve patients 428 and 935) and recurrent (treatment-resistant patients 241 and 972) tumors were obtained from consenting patients as approved by the Hamilton Health Sciences/McMaster Health Sciences Research Ethics Board (REB# 07-366). Brain tumor samples were dissociated in the PBS (Thermo Fisher Scientific, cat# 10010049, Waltham, MA, USA) containing 0.2 Wünsch unit/mL Liberase Blendzyme 3 (Millipore Sigma, cat# 5401119001) and incubated in a shaker at 37 °C for 15 min. The dissociated tissue was filtered through a 70 μm cell strainer (Falcon, cat# 08-771-2) and collected by centrifugation (1500 rpm, 3 min). Red blood cells were lysed using an ammonium chloride solution (STEMCELL Technologies, cat# 07850, Vancouver, BC, CA). GBM cells were resuspended in the Neurocult complete (NCC) media, a chemically defined serum-free neural stem cell medium (STEMCELL Technologies, cat# 05751), supplemented with the human recombinant epidermal growth factor (20 ng/mL; STEMCELL Technologies, cat# 78006), basic fibroblast growth factor (20 ng/mL; STEMCELL Technologies cat# 78006), heparin (2 μg/mL 0.2% heparin sodium salt in the PBS; STEMCELL technologies, cat# 07980), antibiotic-antimycotic (1×; Wisent, cat# 450-115-EL, Montreal, QC, Canada), and plated on ultra-low attachment plates (Corning, cat# 431110, Corning, NY, USA) and cultured as neurospheres and used with minimal culturing (<20 passages).

Neurospheres were plated on polyornithine-laminin coated plates for adherent growth. Adherent cells were replated in low-binding plates and cultured as tumorspheres, which were maintained as spheres upon serial passaging in vitro. Compared to commercially available GBM cell lines which are cultured in mono-layers, patient-derived 3D cultures represent the variety of heterogeneous clones present within patient samples [42].

### 2.4. Western Analysis

Protein extracts were prepared using a lysis buffer (50 mM Tris–HCl, 200 mM NaCl, 0.2% NP-40, 1% Tween-20 (*v/v*)) supplemented with 1 mM NaF, 1 mM sodium vanadate, 50 mM β-glycerophosphate, 2 mM phenylmethylsulfonyl fluoride (PMSF), and Complete protease inhibitor (Roche, Basil, Switzerland). Following quantification using the Bradford assay (Bio-Rad, Hercules, CA, USA), proteins (20 µg per lane) were separated through a Novex 4–12% (*w/v*) Bis-Tris Sodium Dodecyl Sulfate (SDS) polyacrylamide gel (Life Technologies) and transferred onto a nitrocellulose membrane (Bio-Rad). Blots were sequentially immunostained with either anti-RAD51 monoclonal antibody (mAb) (Cell Signaling Technology, cat# 8875, 1:1000, Danvers, MA, USA), anti-PTEN mAb (Cell Signaling Technology, cat# 9188, 1:1000), mouse anti-PAR polyclonal antibody (pAb) (Trevigen, cat# 4335-MC-100, 1:1000, Gaithersburg, MD, USA), mouse anti-PARP1 mAb (Millipore-Sigma, cat# AMAb90959, 1:1000), rabbit anti-p-KAP1 mAb (Cell Signaling Technology, cat# 4127, 1:1000), rabbit anti-KAP1 mAb (Cell Signaling Technology, cat# 4124, 1:1000), rabbit anti-p-ATM^S1981^ mAb (Cell Signaling Technology, cat# 13050, 1:1000), rabbit anti-ATM mAb (Cell Signaling Technology, cat# 2873, 1:1000), rabbit anti-p-53BP1^S1778^ pAb (Cell Signaling Technology, cat# 2675, 1:1000), or rabbit anti-53BP1 pAb (Cell Signaling Technology, cat# 4937, 1:1000), followed by a horseradish peroxidase–conjugated goat anti-mouse (Bio-Rad, cat# 1706516, 1:2000) or a goat anti-rabbit antibody (Bio-Rad, cat# 1706515, 1:2000) and detected using the Clarity chemiluminescence reagent (Bio-Rad) and imaged using an ImageQuant LAS 500 automated chemiluminescence imager. Rabbit anti-actin pAb (Millipore-Sigma, cat# A2066, 1:2000) or rabbit anti-Scythe ab [43] (1:5000) were used as protein-loading controls. Cell treatments for Western analysis are in each respective figure legend. Blots were visualized using a LAS 500 Imager (GE Healthcare). Densitometric analysis of individual protein bands was performed via ImageJ (National Institute of Health, Bethesday, MD, USA). Protein levels were first normalized to a loading control and any concomitant phospho-protein levels were normalized to their non-phosphorylated counterparts.

Control (Ctrl; GM06889) and A–T (ataxia–telangectasia) (GM01526) human lymphoblastoid cell lines (LCL) were obtained from the Coriell Institute for Medical Research (Camden, NJ, USA). LCLs were maintained in the RPMI media supplemented with 15% fetal bovine serum (*v/v*), 1× glutamax, 100 U/mL penicillin, 100 μg/mL streptomycin. Cells underwent 8 Gy irradiation using an RS-2000 (Rad Source Technologies, Inc., Buford, GA, USA), followed by 60 min incubation (37 °C) to mediate cellular DNA damage-mediated signaling/phosphorylation events. Protein extracts were prepared, run, and analyzed as above.

### 2.5. Alkali Comet Assay

Quiescent (non-proliferating) primary astrocytes and MEFs were treated with γ–irradiation (IR, 20 Gy). Following IR treatments, cells were incubated for various times in a drug-free medium at 37 °C (recovery). Cells were trypsinized and suspended in a pre-chilled 1× PBS mixed with an equal volume of 1.2% UltraPure low-melting point agarose (Invitrogen) maintained at 42 °C and immediately layered onto frosted glass slides (Fisher) pre-coated with 0.6% agarose and maintained in the dark at 4 °C for all further steps. Slides were immersed in a pre-chilled lysis buffer (2.5 M NaCl, 10 mM Tris–HCl, 100 mM Ethylenediaminetetraacetic acid (EDTA) (pH 8.0), 1% (*v/v*) Triton X-100, 3% (*v/v*) DMSO, pH10) for 1 h, washed with pre-chilled distilled water (twice for 10 min each), and placed into a pre-chilled alkaline electrophoresis buffer (50 mM NaOH, 1 mM EDTA, 1% DMSO) for 45 min. Electrophoresis was carried out at 95 mA (astrocytes) or 190 mA (MEFs) for 25 min, followed by neutralization in 0.4 M Tris–HCl (pH 7.0). Comets were stained with SYBR Green (1:10,000 in 1× PBS, Sigma) for 10 min. A minimum of 100 comet tail moments were measured using the Comet Assay IV system (Perceptive Instruments) coupled to an Axioskop2 plus microscope (Zeiss) at 200× magnification as previously described [38,41]. All data reflect experiments performed in triplicate (*n* = 3, *N* ≥ 300).

Human glioblastoma cell lines, U87MG, U251MG, and U373MG, were transduced with shRNAs to silence *RAD51* gene expression (or with *Scm* as control) and underwent treatment with the radiomimetic Neocarzinostatin (NCS; Sigma cat# N9162-100UG, St. Louis, MO, USA) at 340 ng/mL for 1 h followed by alkaline comet analysis. Briefly, cells were resuspended in 1% UltraPure low melting point agarose and plated on a 96-well CometSlide (Trevigen; cat# 4253-096-03) and allowed to dry. Slides were incubated in a lysis buffer for 1 h at 4 °C and then placed into an alkaline unwinding buffer (200 mM NaOH, 1 mM EDTA; pH > 13) protected from the light at room-temperature (RT) for 20 min. Slides were then placed into a cold electrophoresis buffer (50 mM NaOH, 1 mM EDTA, 1% DMSO) within an electrophoresis manifold (Trevigen; #4250-050-ES), followed by electrophoresis at 21 V for 40 min. Slides underwent two successive washes with double-distilled water, followed by a 70% ethanol wash and air drying. DNA was stained with SYBR Green (Sigma-Aldrich; cat# S9430; 1:10000 in 1× PBS) for 10 min. A minimum of 300 comet tail moments per experiment per cell line/treatment were measured using a Cytation 5 (Biotek Instruments, Winooski, VT, USA) multi-mode reader with a 2.5× objective capturing a fixed area encompassing ≥ 100 cells per well, with 3 wells being measured for each treatment group in each experimental set (*N* ≥ 300). Gen5 analysis software (Biotek Instruments, Winooski, VT, USA) was used to determine the average length of a comet tail moment. For U87 and U373, *n* = 5 and *N* ≥ 1500; for U251, *n* = 4 and N ≥ 1200.

### 2.6. Quantification of DNA Damage-Induced Nuclear Foci

For the fluorescent labeling of quiescent primary murine cortical astrocytes and MEFs, cells were grown to confluence on glass coverslips, underwent irradiation (+/− recovery at 37 °C) and then fixed with 4% paraformaldehyde (PFA) in PBS for 10 min, followed by three successive washes with 1× PBS, and then permeabilized for 5 min in 0.5% Triton X-100/PBS. Following three successive washes with 1× PBS, cells were immunostained with antibodies diluted in 3% bovine serum albumin (BSA) + 1× PBS; anti-γH2AX (Ser139, mouse, 1:500; Millipore, cat# JBW301) and anti-RAD51 (rabbit, 1:500; Calbiochem), followed by Alexa Fluor 488/555-conjugated secondary antibodies (1:500, Life Technologies, cat# A-21206 and A-31570, respectively), and counterstained with 4′,6-diamidino-2-phenylindole (DAPI). Experiments were performed in triplicate (*n* = 3) with 30 cells measured per experiment (*N* = 90). Xrcc2/p53-double-deficient primary MEFs served as an HRD control and were previously described [44].

GBM cells were seeded at a density of 8000 cells per well in a 96-well black clear flat-bottomed plate (Corning, cat# 07-200-588). The cells were treated with neocarzinostatin (NCS) (140 ng/mL) or media as the vehicle control for 1 h. Cells were then fixed with 4% PFA (in 1× PBS) for 10 min at room temperature followed by a washing step with PBS (2×) and permeabilization with 0.5% Triton X-100 in the PBS for 5 min at room temperature. Following an additional washing step with the PBS (2×), primary antibodies against phospho-53BP1 (S1778) (rabbit, Cell Signaling Technology, cat# 2675S, 1:1000) and RAD51 (mouse, Abcam, cat# ab213, 1:500) were prepared in a 3% BSA (in 1× PBS) and added to the cells at a volume of 50 μL/well for overnight incubation at 4 °C in the dark. Cells were then washed with 1× PBS (2×) and immunostained with Alexa Fluor-488 donkey anti-rabbit and Alexa Fluor-555 donkey anti-mouse dyes (1:500, Life Technologies, cat# A-21206 and A-31570, respectively) prepared in 3% BSA (1× PBS) at a volume of 50 μL/well for 2 h at room temperature in the dark. The cells were then washed with the PBS (1×), fixed with 1% PFA (in 1× PBS) for 10 min, and permeabilized with 0.5% Triton X-100 for 5 min at room temperature, taking care not to expose the assay plate to light. Following another wash step with 1× PBS, the fixed cells were immunostained with an anti-phospho-H2AX (Ser139) antibody pre-conjugated to Alexa Fluor-647 (Biolegend; cat# 93144; 1:500 dilution) for 1 h at room temperature. The cells were washed in 1× PBS (2×) then counterstained with Hoechst 33342 (0.2 μg/mL in the PBS; Life Technologies, cat# H1399) to visualize cell nuclei. Image acquisition and analysis were carried out using a Cytation 5 cell imaging multi-modal reader and the Gen5™ Imaging Software (Biotek Instruments). Experiments were performed in triplicate (*n* = 3) with 100 cells measured per experiment (*N* = 300).

### 2.7. Cell Viability Assays

Cell viability was determined by counting the cells stained with the Hoechst 33342 nucleic acid stain solution at 24 h increments over a 96 h period. Briefly, glioblastoma cell lines U87MG, U251MG, and U373MG transfected with shRNAs to RAD51 or Scm control, were seeded at a density of 1000 cells/well in a 96-well plate. Wells were treated with either DMSO (control) or the PARPi olaparib (AZD2281, Ku-0059436, Apexbio, cat# A4154) at a concentration of 1 μM and the cells were incubated at 37 °C (with 5% CO_2_) for 96 h. At each 24 h interval, Hoechst 33342 (Life Technologies, cat# H1399) at a concentration of 0.2 μg/mL was applied to a subset of wells and the subsequently-labeled cell nuclei were counted and analyzed via Cytation 5 and Gen5. Experimental assays were repeated in triplicate with 3 wells per cell treatment/time-point per experiment (n = 3, N = 9). Cell growth rate was determined relative to the baseline cell count performed upon cell seeding (Day 1). Statistical analysis was performed using the 2-way ANOVA using the Tukey’s post hoc test with no correction.

### 2.8. Gene Expression Analyses According to the PTEN Status (TCGA and SKS Datasets)

Mutational (whole exome sequencing) and transcriptomic (mRNA expression, RSEM values) data of 145 glioblastoma tissue specimens were gathered from The Cancer Genome Atlas (TCGA) PanCancer Atlas study [45] using cBioPortal [46,47]. Samples with one or more amino acid changes (missense or frameshift mutant) in the *PTEN* coding sequence were categorized as mutant/deletion (mut/del), whereas all remaining samples were considered wild-type (WT). Transcriptomic RSEM values for genes of interest (*PTEN, RAD51, RAD51B, RAD51C, RAD51D, XRCC2, XRCC3* and *DMC1*) were matched with the *PTEN* status and compared between WT and mut/del.

Patient-derived primary glioblastoma specimens were obtained, processed, cultured, and enriched for glioblastoma brain tumor initiating cells (BTICs) as previously described [48]. RNA was extracted from each BTIC line using the RNeasy Kit (Qiagen) or Total RNA Purification Kit (Norgen). Samples were submitted for mRNA-Seq (SKS dataset) at the Donnelly Sequencing Centre at the University of Toronto (http://ccbr.utoronto.ca/donnelly-sequencing-centre) or at MedGenome Inc. (https://diagnostics.medgenome.com/research-services). Further details about sequencing and data analysis can be requested from Dr. Sheila K. Singh (ssingh@mcmaster.ca). Variant calls for PTEN were obtained using the Genome Analysis Toolkit (GATK) best practices pipeline [49,50]. Samples with one or more amino acid changes (missense or frameshift mutant) in the *PTEN* coding sequence were categorized as mutant/deletion (mut/del), whereas all remaining samples were considered wild-type (WT). Normalized transcript read count values for genes of interest (*PTEN, RAD51, RAD51B, RAD51C, RAD51D, XRCC2, XRCC3* and *DMC1*) were matched with the *PTEN* status and compared between WT and mut/del.

## 3. Results

### 3.1. RAD51 Expression and HR Activity Are Not Impaired in PTEN-Deficient Primary Cells

**Figure 1:**
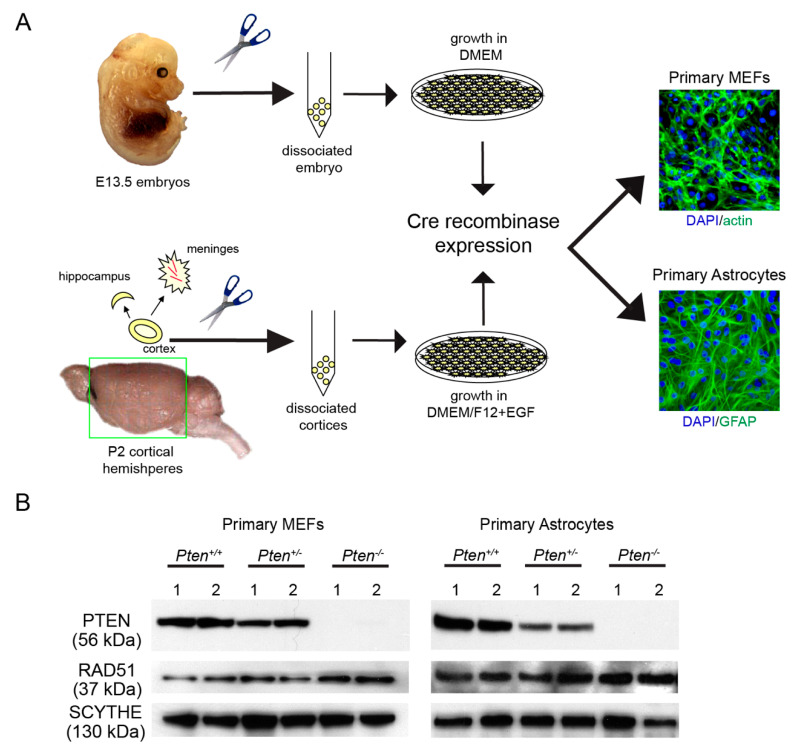
Establishing Pten-deficient primary mouse cells for Rad51 protein analysis. (**A**) Schematic of a protocol to isolate and establish primary murine *Pten*-null (and controls) cortical astrocytes (P2 newborns) and MEFs (E13.5). Following culture establishment, primary cells were transduced with MSCV-creGFP retroviral particles and quantified for GFP positivity. Following expansion of Cre+/GFP+ cells, genomic analysis confirmed Cre recombination followed by immunofluorescence analysis, using phalloidin for MEFs or an anti-glial fibrillary acidic protein (GFAP) antibody for astrocytes to confirm cell type purity, respectively. Only cultures with >90% GFAP/phalloidin positivity were used for prospective biochemical/cellular analyses. (**B**) Western analysis of primary murine *Pten*-null (and controls) astrocytes and MEFs. Protein extracts were prepared using Cre-modified primary cell cultures and underwent Western analysis to confirm the Pten expression status and corresponding RAD51 levels. Consistent with genomic analyses, cultures derived from *Pten* heterozygotes (*Pten^+/−^*) displayed reduced Pten protein expression compared to the wild type (Pten^+/−^), while those from the *Pten* knockout (*Pten^−/−^*) did not display Pten protein expression. RAD51 protein levels remain unchanged regardless of the Pten expression status. Anti-Scythe ab was used as a protein loading control [43].

**Figure 2:**
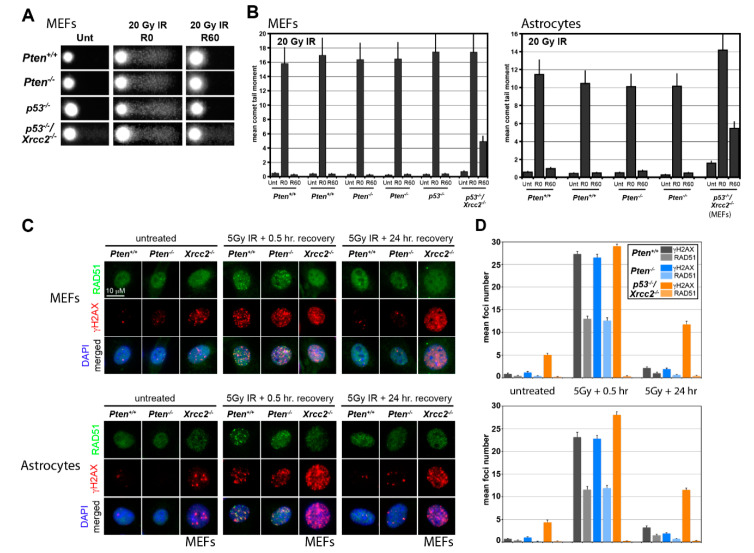
DNA damage analysis of Pten-deficient primary murine mouse embryonic fibroblasts (MEFs) and astrocytes. (**A**) Representative images from the alkaline comet analysis of primary *Pten^+/+^, Pten^−/−^, p53^−/−^* and *Xrcc2^−/−^; p53^−/−^* MEFs following 20 Gy irradiation (R0) and 60 min post-irradiation recovery (R60). Although comet tail lengths appear equivalent in all genetic cell subtypes following radiation, no residual tails remain in *Pten^+/+^, Pten^−/−^* and *p53^−/−^* MEFs 60 min following radiation, while *Xrcc2^-/-^; p53^-/-^* MEFs show a residual tail. (**B**) Bar graphs represent mean comet tail moments plus the standard error of means (s.e.m.) derived from the alkaline comet analysis on primary MEFs and astrocytes. Experiments were repeated in triplicate (*n* = 3), where a minimum of 100 cells were measured per line/treatment in each experiment; *N* ≥ 300 total independent comet tail moments were measured per line/treatment. Wild-type and *Pten^−/−^* MEFs and astrocytes show equivalent repair activity following irradiation. Similar to the wild-type and *Pten^−/−^* cells, *p53^-/-^* cells show no apparent DNA repair defect, while *Xrcc2^−/−^, p53^−/−^* cells display delayed HR-dependent repair following DNA strand break damage [44]. (**C**) Representative fluorescent γH2AX and RAD51 foci micrographs derived from primary *Pten^+/+^, Pten^−/−^* and *Xrcc2^−/−^; p53^−/−^* MEFs and astrocytes following irradiation and 24 h post-irradiation recovery. In untreated *Pten^+/+^* and *Pten^−/−^* cells, both γH2AX and RAD51 foci numbers are minimal, while the γH2AX foci are apparent in *Xrcc2^−/−^, p53^−/−^* cells. Following irradiation, *Pten^+/+^* and *Pten^−/−^* cells develop γH2AX and RAD51 foci, while *Xrcc2^−/−^, p53^−/−^* cells only develop γH2AX foci. RAD51 foci fail to form in *Xrcc2^−/−^, p53^−/−^* cells regardless of treatment despite expression of the RAD51 protein consistent with HR deficiency. Following 24 h of post-irradiation recovery, γH2AX and RAD51 foci are absent in *Pten^+/+^* and *Pten^−/−^* cells, indicating repair of the radiation-induced damage, while pervasive γH2AX foci remain in *Xrcc2^−/−^, p53^−/−^* cells indicating unrepaired DNA strand break damage. (**D**) Bar graphs represent mean γH2AX and RAD51 foci per cell (foci/cell) quantification data of primary MEFs and astrocytes following 5 Gy radiation and recovery 24 h post-radiation. Experiments were repeated in triplicate (*n* = 3), where a minimum of 30 cells were measured per treatment; total *N* ≥ 90 independent cells measured per line/treatment. Error bars represent the standard error of means (s.e.m.).

To investigate the link between PTEN and RAD51, multiple approaches were undertaken. Primary *Pten*-null (and corresponding wild-type) mouse embryonic fibroblasts (MEFs) and astrocytes were established (Figure 1A). Western analysis confirmed the Pten expression status, which was consistent with corresponding *Pten* genotypes. Subsequent immunostaining revealed Rad51 expression (Figure 1B) regardless of the *Pten* genotype, indicating that loss of Pten does not affect Rad51 expression. To determine if a functional relationship exists between the PTEN expression and DNA repair activity, cells were irradiated and analyzed via the alkaline comet analysis. In both primary fibroblasts and astrocytes, cells incurred similar levels of DNA damage following irradiation, irrespective of Pten expression (Figure 2). One hour following damage, both the *Pten^-/-^* and *Pten^+/+^* (wild-type) cell populations show complete DNA repair, indicating that DNA repair activity is equivalent in wild-type and *Pten*-null cells. In comparison, comet analysis of *Xrcc2*-null cells, which normally express the Rad51 protein [51], display higher comet tail moment values than wild-type or *Pten^-/-^* cells one hour following irradiation (Figure 2A,B), confirming the deficiency in homologous recombination (HR) [44]. Complementary DNA repair assays examining γH2AX and RAD51 foci, indicators of DNA double-stranded breaks and HR activity, respectively, yielded similar results. In both fibroblasts and astrocytes, wild-type and *Pten^-/-^* cells accumulated equivalent numbers of γH2AX foci following irradiation (Figure 2C,D). Moreover, all cells displayed pervasive RAD51 immunostaining accompanied by formation of RAD51 immunopositive foci, indicating active HR activity. In contrast, *Xrcc2*-null cells are RAD51-immunopositive but fail to form RAD51 foci in spite of the presence of endogenous and radiation-induced γH2AX foci. Combined, these DNA damage repair data indicate that PTEN-deficiency does not impact RAD51 expression or disrupt RAD51/HR-dependent DNA damage repair.

### 3.2. PTEN-Deficient Cancer Lines Express RAD51 and Exhibit Robust DNA Repair Activity

**Figure 3:**
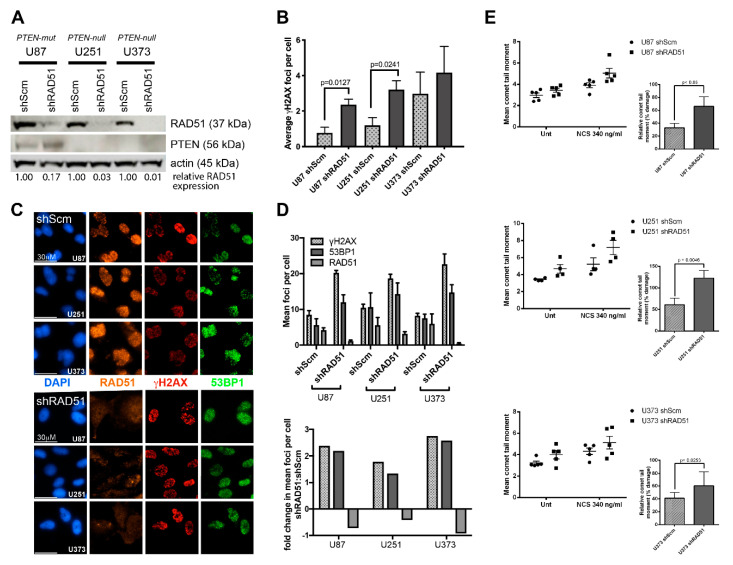
DNA repair analysis of PTEN-deficient glioblastoma multiforme (GBM) cell lines. (**A**) Western analysis of PTEN-deficient GBM cell lines stably expressing *shRAD51* or *shSCM* (control). RAD51 knockdown in shRAD51-expressing GBM lines achieved ≥ 83% reduction in the RAD51 protein relative to *shSCM*-expressing GBM counterparts (83% in U87MG; 97% in U251MG; and 99% in U373MG). Immunostaining with anti-PTEN antibody confirms the reduced expression of mutant PTEN in U87 and loss of PTEN in U251 and U373. (**B**) Untreated *shRAD51*-GBM cells develop higher basal γH2AX foci (DNA damage) in comparison to the untreated *shSCM*-GBM counterparts. (**C**) Representative immunofluorescent micrographs and bar graphs quantifying γH2AX, RAD51, and 53BP1 (phospho-53BP1^S1778^) foci among *shSCM*- and *shRAD51*-GBM cells following neocarzinostatin (NSC)-induced DNA double-strand breaks. Cellular treatment with NSC (140 ng/mL) induces γH2AX and 53BP1 foci in both *shSCM*- and *shRAD51*-GBM cells with only *shSCM*-GBM cells showing concomitant RAD51 foci, while *shRAD51*-GBM cells fail to display RAD51 nuclear foci or signal. Scale bar represents 30 μm. (**D**) Bar graphs quantify the mean number of DNA double strand break (DSB)-dependent foci in all *shSCM*- and *shRAD51*-GBM cells following 140 ng/mL NSC treatment. Only *shSCM*-GBM cells show formation of RAD51 foci, while *shRAD51*-GBM cells do not. However, *shRAD51*-GBM cells display approx. 1.5–2.5× more 53BP1 foci compared to *shSCM*-GBM cells. Statistical analysis was performed using the unpaired Student’s t-test (U87 and U251, *n* = 4; U373, *n* = 3). (**E**) The effect of RAD51 depletion in glioblastoma tumor cell lines shown by the alkaline comet assay. Cells were exposed to NCS (340 ng/mL) for 1 h and the extent of NCS-induced DNA damage was quantified using the alkaline comet assay. (Top graphs) Mean comet tail moment values of glioblastoma cells following NCS treatment and (Bottom graphs) DNA damage percentage relative to the untreated control. Increased DNA damage was observed in *RAD51*-knockdown cells in comparison to the *SCM* control as a result of the defective HR that is expected only in RAD51-deficient cells. A slight increase in DNA damage was also observed in all untreated RAD51-deficient GBM cells in comparison to the *SCM* counterpart. Statistical analysis was performed using the unpaired Student’s t-test (U87, U373, *n* = 5, *N* ≥ 1500; U251, *n* = 4, *N* ≥ 1200).

Although *PTEN* loss is a frequent genetic driver during tumorigenesis, a vast majority of tumors may accumulate mutation(s) to one or more DNA repair genes resulting in aberrant DNA repair activity [52,53]. We examined whether PTEN-deficient human GBM cell lines U87MG, U251MG, and U373MG display dysfunctional DNA double-strand break repair, particularly, RAD51-dependent HR activity. These cells harbor previously described *PTEN* mutations resulting in either exon deletion (U87MG: PTEN-mut) or premature termination (U251MG and U373MG: PTEN-null), thus inactivating PTEN function [54]. To quantify relative RAD51 expression and HR/DNA repair activity in these PTEN-deficient lines, GBM cells underwent lentiviral transduction and puromycin selection in order to generate stable cell lines expressing shRNAs targeting *RAD51* or scrambled control (*SCM*) (Figure S1 and Figure 3A). While RAD51 protein is highly expressed in all three PTEN-deficient *shSCM*-GBM lines (control), ≥83% of the RAD51 protein is reduced in *shRAD51*-GBM (RAD51-deficient) lines. Likewise, DNA damage analysis of all stable *shRAD51*-expressing GBM lines demonstrate up to a 4-fold increase in endogenously occurring γH2AX foci compared to *shSCM*-GBM control lines (Figure 3B). Furthermore, induction of DNA double-stranded breaks with the NCS treatment (140 ng/mL for 1 h at 37 °C) results in a ~2 fold increase in γH2AX and 53BP1 foci in shRAD51-expressing cells compared to the *shSCM*-GBM counterparts (Figure 3C,D). However, RAD51 foci are only present in *shSCM*-GBM cells and are concomitantly increased with the NCS treatment but are absent in *shRAD51*-GBM cells regardless of DNA damage levels (Figure 3C,D). Similarly, the alkali comet analysis shows a significant accumulation of DNA damage in all NCS-treated *shRAD51*-GBM (RAD51-deficient) lines compared to NCS-treated *shSCM*-GBM control lines (*p* ≤ 0.05) (Figure 3E). Analysis of an unrelated malignant PTEN-deficient human B-cell line, BJAB, shows robust RAD51 expression and intact ataxia telangiectasia mutated (ATM)- and KRAB-associated protein 1 (KAP1)-dependent activation of DNA damage responses (Figure S2) [55]. Combined, these data indicate that PTEN-deficient GBM cells retain both RAD51/HR- and 53BP1/NHEJ-dependent DNA DSBR activities, while induced HRD results in enhanced accumulation of DNA damage and concomitant compensatory NHEJ activity.

**Figure 4:**
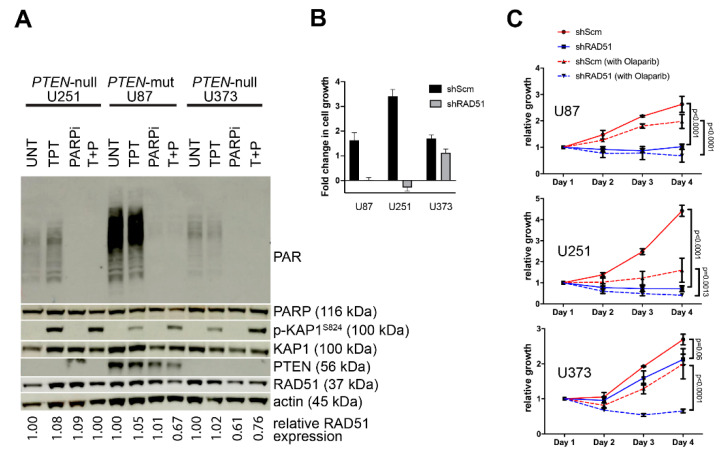
Cell proliferation analysis in PTEN-deficient GBM cell lines following treatment with PARP inhibitor (PARPi) olaparib. (**A**) GBM cells underwent treatment with olaparib (5 μM pre-treatment for 30 min followed by 1 h at 37 °C) and/or topotecan (5 μM for 1 h at 37 °C). Western analysis of the topotecan-treated cells displayed the DNA damage-induced ATM-dependent KAP-1 phosphorylation (Figure S2) and enhanced PARylation, while the olaparib treatment alone failed to induce KAP-1 and suppressed the basal PARylation and dual treatment results in KAP-1 phosphorylation in the absence of PARylation. (**B**) Cells that undergo *shRAD51*-mediated knockdown of RAD51 display significant reduction in cellular growth. (**C**) While olaparib treatment (1 μM) of *shSCM*-GBM control lines moderately impacts the GBM cell growth (compare the red solid line to the hashed line), olaparib treatment of *shRAD51*-GBM lines shows cell loss (compare the blue solid line to the hashed line). The statistical analysis was performed using the 2-way ANOVA using the Tukey’s post hoc test with no correction.

Recent studies suggest that PTEN-deficient tumors are responsive to PARPi due to purported RAD51/HR loss similar to PARPi-induced synthetic lethality in tumors that feature BRCA/HR-deficiency [32]. Although we find that RAD51 expression remains intact in PTEN-deficient cells, we examined whether PTEN-deficiency could result in enhanced DNA damage and sensitivity to PARPi. PTEN-deficient GBM cells underwent treatment with olaparib (AZD-2281, Ku-0059436), a clinically-relevant PARPi regularly used in the treatment of BRCA-deficient breast, ovarian, and endometrial cancer [27]. Despite effective inhibition of **P**oly **A**DP-**R**ibosylation (PARylation) (Figure 4A), olaparib treatment failed to induce a discernable DNA damage response in GBM cells, whereas mono- or co-treatment with topotecan (TPT), a DNA strand break-inducing Topoisomerase-1 inhibitor, stimulated ATM-dependent KAP-1 phosphorylation, indicating DNA damage signaling activation (Figure 4A) [55].

All three GBM cell lines underwent cellular viability analysis to determine whether PTEN-deficient cancer cells have enhanced sensitivity to PARPi (Figure 4B,C). Olaparib treatment of both *shSCM*- and *shRAD51*-GBM lines was used to compare the specific requirement of RAD51 expression in PARPi-mediated growth suppression. The three GBM cell lines showed varying sensitivity to olaparib, which was not related to endogenous RAD51 levels. Interestingly, expression of *shRAD51* and the resultant loss of the RAD51 protein (Figure S1) reduces cellular growth independently of PARPi treatment (Figure 4B,C). However, in U373 cells where RAD51 depletion causes a modest decrease in growth, olaparib co-treatment completely ablates cell growth in the other shRAD51 lines, while only moderately suppressing cell growth in all *shSCM* control lines (Figure 4C). The apparent difference in response with the *shRAD51*-U373 line compared to the *shRAD51*-U87 and *shRAD51*-U251 lines is likely due to the previously described reduced proliferative rate and colony-forming capacity of U373 compared to U251 and U87 [56]. As HR is primarily associated with proliferation, the impact of *shRAD51*-induced HRD in U373 would be expected to be muted compared to those of U87 and U251. Combined, these data demonstrate that PTEN deficiency has no role in mediating RAD51-dependent sensitivity to olaparib or in disrupting HR-dependent DNA damage signaling and repair responses which would otherwise synergize with PARPi.

**Figure 5:**
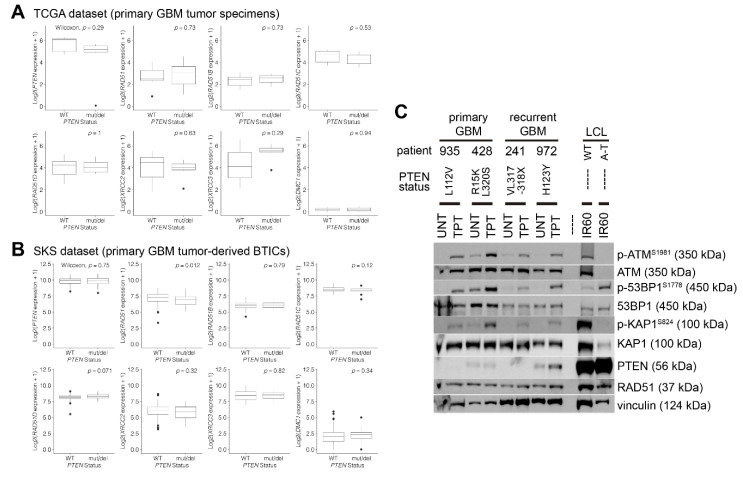
Expression analysis of RAD51 and its paralogs in *PTEN*-mutated/deficient GBM. (**A**) Expression analysis of *RAD51* and its paralogs stratified by the *PTEN* status from The Cancer Genome Atlas (TCGA) dataset. Box-and-whisker plots of log2-transformed gene expression levels in glioblastoma tissue specimens from TCGA sorted by the *PTEN* mutational status denoted as wild-type (WT; *n* = 95) or mutant/deletion (mut/del; *n* = 50). An unpaired Wilcoxon rank-sum test was conducted between *PTEN* WT and mut/del expression values for each gene, resulting in the displayed *p* values. (**B**) Expression analysis of *RAD51* and its paralogs stratified by the *PTEN* status from patient-derived GBM brain tumor initiating cells (BTICs). Box-and-whisker plots of log2-transformed gene expression levels in BTICs from the SKS dataset sorted by the *PTEN* mutational status denoted as wild-type (WT; *n* = 7) or mutant/deletion (mut/del; *n* = 6). An unpaired Wilcoxon rank-sum test was conducted between *PTEN* WT and mut/del expression values for each gene, resulting in the displayed *p* values. (**C**) Western analysis of the BTICs derived from primary and recurrent GBM patients. Each BTIC line is derived from a GBM patient with an independent *PTEN* mutation (*PTEN* status) predicted to abrogate the PTEN protein stability/function based on the Gene Analysis Toolkit (GATK) sequence analysis (https://gatk.broadinstitute.org/hc/en-us; Broad Institute, Cambridge, MA, USA) and the previous studies [58]. RAD51 is expressed in all *PTEN*-mutant/deficient primary GBM BTICs, while the DNA damage generated by topotecan (TPT; 10 μM at 37 °C for 2 h) induces robust phosphorylation of 53BP1 (p-53BP1^S1778^) indicating active cellular non-homologous end joining (NHEJ) activity. The presence of phosphorylated ATM (p-ATM^S1981^) and KAP1 (p-KAP1^S824^) is indicative of induction of the DNA damage response. Irradiation of immortalized lymphoblastoid cells (LCLs) from normal (WT) and ataxia–telangiectasia (A–T) patients serve as ATM-proficient and -deficient control lines, respectively, to track ATM/KAP1-mediated DNA damage signaling.

To determine whether expression of RAD51 or its paralogs is impacted by PTEN-dysfunction in primary tumors, we performed an RNA expression analysis amongst *PTEN*-mutant/deleted tumors derived from glioblastoma patients. Analysis of The Cancer Genome Atlas (TCGA) dataset (Figure 5A) of glioblastoma tissue specimens and the SKS dataset (Figure 5B) of patient-derived glioblastoma brain tumor initiating cells (BTICs) revealed no notable differential expression of RAD51 or its paralogs in *PTEN*-mutated or wild-type samples. A minor decrease in RAD51 expression was observed in *PTEN*-mutant samples from the TCGA dataset as compared to the wild type (*p* = 0.012), although this difference was nominal (<7%). Furthermore, we performed protein expression and DNA damage response signaling analyses on 2 primary (treatment-naïve) and 2 recurring (treatment-resistant) GBM patient-derived BTIC lines (Figure 5C). These cells recapitulate the key morphological, architectural, and expression features that are present in the initial clinically presented GBM tumor while retaining their self-renewal potential and capability to form tumors in vivo [57]. Each patient-derived BTIC line contains an independent PTEN mutation predicted to abrogate the PTEN protein function and/or stability [49,50,58]. In all cases, RAD51 expression was apparent and topotecan-induced DNA damage resulted in robust ATM-dependent activation of KAP1 (S824) and phosphorylation of 53BP1 (S1778). Combined, these patient tumor data indicate that primary GBM tumors do not incur PTEN-dependent expression changes neither in RAD51 nor its paralogs and retain functional DNA damage signaling, including HR and NHEJ activity.

## 4. Discussion

“Synthetic lethality” (and the related “synthetic sickness”) in anti-cancer therapy arose from success in killing cancer cells by specifically targeting compensatory mechanisms, pathways, or activities that usually allow for tumor cell survival and growth despite the loss of the functionally important parallel cell growth/survival pathways [59,60]. Perhaps the best known example of such phenomenon involves mutations and/or low expression of *BRCA1/2*, whereby HR deficiency sensitizes tumor cells to inhibit PARP1, a critical mediator of the corresponding DNA single-strand break repair pathway [17,61,62,63]. This finding enabled the use of the first clinically relevant chemical inhibitor of PARP, olaparib, which spares normal HR-proficient cells while specifically targeting HRD tumor cells to undergo enhanced DNA damage-induced cell death [64,65]. As a corollary, a functional loss of other proteins involved in HR fidelity and DNA DSBR is thought to confer tumor cells a “BRCAness” phenotype [66,67,68,69,70]. Clinical olaparib use in BRCA-deficient breast, ovarian, and endometrial cancers is well-documented and has spurred recent trials in prostate, lung, and colon cancers that feature HRD [27,37,69]. Furthermore, this clinical success is the basis of numerous biomedical studies that seek to link other known tumorigenic mutations to the DNA repair function, either directly or indirectly, to enable wider therapeutic utilization of current and next generation PARPis [23,71]. Amongst the more controversial is the finding that PTEN associates with E2F-1 to regulate the *RAD51* transcription, a key component of the tripartite BRCA1/2 HR DNA repair complex [30]. Subsequent studies suggested that PTEN-deficient tumor lines acquire HRD and in turn a BRCA-like phenotype, thus sensitizing these cells to olaparib [32,33,72,73]. Considering that *PTEN* is one of the most frequently mutated genes in cancer [3,4], with 40–80% of most human tumors acquiring somatic *PTEN* mutation, deletion, and/or loss of heterozygosity [74,75], use of PARPi to treat these tumors would represent a profound therapeutic advancement. However, more recent studies, including our findings, demonstrate the opposite; that there is no functional link between *PTEN* and *RAD51* co-expression nor any basis for the use of PARPi to ablate PTEN-deficient tumors as *PTEN* loss does not uniformly predict response to olaparib. In our study, we employ multiple types of PTEN-deficient cell models, both primary and tumorigenic, to demonstrate that: (i) expression of PTEN has no bearing on the expression of RAD51 or its paralogs, (ii) PTEN loss does not impart cellular HRD, (iii) PTEN expression has no effect on cellular DNA strand break repair rates, and (iv) PTEN loss does not synergize with PARPi. Consistent with our findings, studies have demonstrated that RAD51 expression and foci formation remain unchanged in other PTEN-deficient tumor cell lines (isogenic HCT116/*PTEN^−/−^*, PC-3, and U251MG), primary astrocytes, RNAi studies (*shPTEN*-expressing H1299), comparative prostatic tumor xenograft analyses (*PTEN^+/+^* 22RV1; *PTEN^+/−^* DU145; and *PTEN^−/−^* PC3), and human tumor tissue microarrays [29,34,35,76]. Non-malignant primary *Pten* wild-type, heterozygote, and knockout astrocytes and MEF cells all display robust RAD51 expression and HR-dependent RAD51 foci formation in response to DNA damage regardless of the *Pten* status. Similarly, we find that PTEN-deficient glioblastoma lines show pronounced RAD51 expression and form HR-responsive RAD51-positive and NHEJ-responsive 53BP1-positive DNA damage-induced foci. Consistent with our data, Miyasaka and associates found that RAD51 levels were expressed broadly and IR-induced γH2AX and RAD51 foci formation was uniform amongst a set of 16 PTEN-proficient and deficient endometrial cell lines [77].

It has been suggested that the apparent discordance in co-expression amongst these findings and other reports may reflect cell type-specific differences involving PTEN/RAD51 [35]. Our study interrogates this assertion by comparing primary versus malignant and neural versus non-neural cell types with the identical conclusion: that RAD51 expression is independent from the PTEN status irrespective of the cell type. Furthermore, RAD51 expression is apparent in replicating (tumor lines) or quiescent (contact-inhibited primary astrocytes/MEFs) PTEN-deficient lines. McEllin et al. suggested that PTEN-associated HRD is not mediated by the loss of RAD51 expression, but rather via reduced expression of the RAD51 paralogs *RAD51B, RAD51C,* and *RAD51D* [29]. Although these analyses were strictly performed in primary murine *Pten*-null astrocytes, our RNA expression analysis in human GBM patient-derived primary tumors and outgrowing BTICs clearly demonstrates that the expression of RAD51 and its paralogs remain unchanged regardless of *PTEN* status.

Although two studies show that PTEN-deficient endometrial tumor cells are not sensitive to PARPi monotherapy [37,77], we observe that PTEN-deficient GBM cell lines are somewhat attenuated with olaparib treatment; observations more consistent with the studies performed on PTEN-deficient prostate cancer cell lines [35]. Furthermore, we show that cell sensitization to olaparib appears to be replication-dependent, as we find that non-replicating cells do not show olaparib sensitivity (data not shown), similar to Gupta et al. [36]. Importantly, only upon *shRAD51* co-expression does olaparib treatment enhance DNA damage and completely ablate PTEN-deficient tumor cell survival. Similarly, Turchick et al. [78] showed that PTEN-deficient tumor cells could only be sensitized upon internalization of a cell-penetrating anti-RAD51 antibody. Combined, these data underscore the functional nature of RAD51-mediated HR activity in PTEN-deficient cells, which mediate cell survival upon inhibition of cellular PARylation.

Our study reinforces the apparently autonomous nature of RAD51 expression and functional HR activity within PTEN-deficient cells, which clearly diverges with the previous studies that originally suggested a PTEN/RAD51 relationship [30,32]. One reason may be the differing origins of Pten-deficient mouse lines [39,79,80] and methods used to develop *Pten*-null MEFs [40,41,79]. However, our data demonstrating that RAD51 expression and HR activity are similar amongst both primary *Pten^-/-^* MEFs and astrocytes suggest that this divergence is likely due to the inherent differences amongst the two genetic *Pten* mouse models. It is noteworthy that cre recombinase expression in our conditional *Pten* (*Pten^LoxP/LoxP^*) astrocyte and MEF cell lines results in deletion of exons 4 and 5, while other murine Pten-Rad51 analyses utilized a conditional *Pten* mouse line with loxP sites flanking only exon 5. Furthermore, McEllin and associates utilized a compound *Pten-Ink4a/Arf* double knockout line [29], which complicates these conclusions as this same group previously published that *de novo* DNA damage induced in Ink4a/Arf single knock-out mice promotes DNA double-strand break-mediated high-grade gliomas [81], which may suggest synergism between PTEN and INK4a/ARF in regulating RAD51 paralog-mediated expression and DNA repair. Future genetic and biochemical comparison of these *Pten* lines may be required to resolve these issues.

The conflicting PTEN-deficient tumor cell data in these studies may be due to the inherently complex genomic and biochemical landscape of tumors, which can accumulate multiple genetic lesions (in addition to PTEN loss), and heterogeneity, but also multiple genetic vulnerabilities, pleotropic roles, and microenvironmental changes throughout tumorigenesis, anti-tumor therapy, and recurrence [75,82]. The PTEN-deficient tumor cells originally used in the findings described by Mendes-Pereira et al. (*PTEN^-/-^* HCT116: MLH1-deficiency) [32] and Shen et al. (PTEN-deficient PC3-AR: PARP1^S383Y^) [30] have since been shown to contain additional genetic lesions resulting in RAD51-independent HRD and enhanced cell sensitivity [83,84,85]. Furthermore, veliparib treatment (another type of PARPi) of PTEN-null PC3-AR prostate cancer mouse xenografts has been shown to enhance RAD51 expression and HR activity under hypoxic conditions and reduce chemotherapy-mediated DNA damage and cell apoptosis [86]. However, further confounding this issue are the recent findings of Ma et al. who showed that in a singular PTEN-deficient cell line (U87MG), RAD51 is functionally expressed, yet these cells display pronounced DNA repair deficiency following radiation-induced DNA damage [87]. Further, they present data showing that radiation therapy can induce fibroblast growth factor receptor (FGFR) family-mediated PTEN^Tyr-240^ phosphorylation and co-recruitment of Ki-67 and RAD51 to chromatin in PTEN-proficient GBM, thus enhancing DSBR activity and radioresistance in GBM patient-derived stem-like cells (GSCs). Although conflicting, their study suggests that contextual modifications of the PTEN protein in tumors may regulate RAD51-mediated HR activity, possibly via FGFR-TACC fusions that accumulate in subsets of PTEN-positive/WT tumors [88]. Our study is unique and definitive as we employ a multitude of PTEN-deficient normal and tumorigenic models to unequivocally demonstrate that RAD51 expression and HR DNA repair activity remain unaffected by PTEN dysfunction. These include *PTEN*-null/mutated primary murine cells (MEFs and astrocytes), human tumor cell lines (U87, U251, U373, and BJAB), and numerous patient-derived glioblastoma tumor specimens and BTICs.

Our findings are further reinforced by the striking lack of clinical evidence supporting therapeutic use of PARPis in the treatment of PTEN-deficient tumors despite the wide breadth of clinical trials [27,28]. In a phase 1 dose escalation trial examining BRCA mutations and sporadic prostate cancer patients treated with a next generation PARPi niraparib, stabilization of the disease occurred in 43% of patients; however, no correlation was observed between PTEN loss and anti-tumor activity [89]. Only a single case report has been published showing a complete remission of a PTEN-null sarcomatoid prostate cancer following olaparib treatment [90]. However, *RB* loss, *TMPRSS2-ERG* rearrangement, and *TP53* alterations were also noted in this patient’s tumor. A more detailed delineation of additional genetic vulnerabilities amongst individual PARPi-responsive PTEN-deficient tumors may reveal additional mechanistic interactions with the PARP-mediated DNA repair [82], thus invoking additional targets for clinical use in combination therapy [91]. In fact, PTEN-deficient endometrial tumor cells are shown to only be sensitive to the PARPi-PI3Ki combination therapy, but not to the PARPi monotherapy [37], while co-inhibition of both RAD51 and ATR is required to synergize ablation of PTEN-deficient melanoma and glioma cells [78], further underscoring the independence of RAD51/HR from the *PTEN* status. In the context of our findings and the apparent lack of clinical evidence, future clinical use of PARPi in the treatment of tumors should be discontinued absent a clear indication of HRD independently of *PTEN* mutational status.

## 5. Conclusions

The identification of HRD mutations in tumors has heralded widespread use of PARPis in a targeted therapeutic approach. Recent findings indicating that PTEN-deficient tumors suppress RAD51 expression and HR activity have been met with controversy, as recent studies have failed to recapitulate these results. Resolving this conflict is paramount due to the ensuing impacts to clinical care and patient trials. Our study demonstrates that RAD51 expression, HR DNA repair activity, and RAD51 foci formation remain intact and unaffected in PTEN-deficient normal (both neural and non-neural) and tumorigenic cells. We further show that *PTEN* loss does not synergize with PARPi. Our findings underscore the functional independence of the *PTEN* mutational status in RAD51-mediated HR and advocate discontinuance of PARPi treatments in PTEN-deficient tumors that do not feature HRD.

## Supplementary Materials

The following are available online at https://www.mdpi.com/2072-6694/12/11/3178/s1: Figure S1: Western analysis of stable *shRAD51*-GBM lines. Combinations of constructs encoding three independent *shRAD51* sequences were lentivirally transduced into U251MG, U87MG, and U373MG, followed by puromycin selection to establish stable *shRAD51*-GBM cell lines. Compared to stable *shSCM*-expressing control lines, the Western analysis using anti-RAD51 antibody revealed that the SIGMA Mission TRCN0000329686 (asterisk) shRAD51 expression construct yields significant reduction in the RAD51 protein within all three GBM cell types. Figure S2: RAD51 is expressed in the PTEN-deficient Burkitt’s lymphoma cell line, BJAB. Irradiation of these cells (10 Gy) irrespective of PARP inhibition induces normal DNA damage responses, including ATM-dependent KAP-1 phosphorylation. Irradiation of immortalized lymphoblastoid cells (LCLs) derived from normal (WT) and ataxia–telangiectasia (A–T) patients serves to obtain ATM-proficient and -deficient control lines, respectively, to track ATM-mediated DNA damage signaling.
