# Supplementary material for: RAD51-Mediated DNA Homologous Recombination Is Independent of PTEN Mutational Status
Source: Cancers (Basel). 2020 Oct 29;12(11):3178. doi: 10.3390/cancers12113178 (PMC7693555; doi:10.3390/cancers12113178)
Supplement: Supplementary file 1 [file cancers-12-03178-s001.pdf]

Supplemental Materials

RAD51-Mediated DNA Homologous Recombination Is Independent of *PTEN* Mutational Status

Asha Sinha, Ali Saleh, Raelene Endersby, Shek H. Yuan, Chirayu R. Chokshi, Kevin R. Brown, Bozena Kuzio, Tiina Kauppinen, Sheila K. Singh, Suzanne J. Baker, Peter J. McKinnon and Sachin Katyal

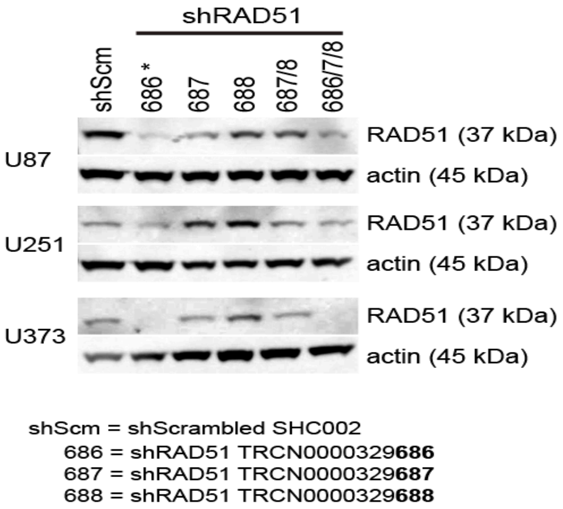

Figure S1. Western analysis of stable *shRAD51*-GBM lines. Combinations of constructs encoding Table 51. sequences were lentivirally transduced into U251MG, U87MG, and U373MG, followed by puromycin selection to establish stable *shRAD51*-GBM cell lines. Compared to stable *shSCM*-expressing control lines, the Western analysis using anti-RAD51 antibody revealed that the SIGMA Mission TRCN0000329686 (asterisk) *shRAD51* expression construct yields significant reduction in the RAD51 protein within all three GBM cell types.

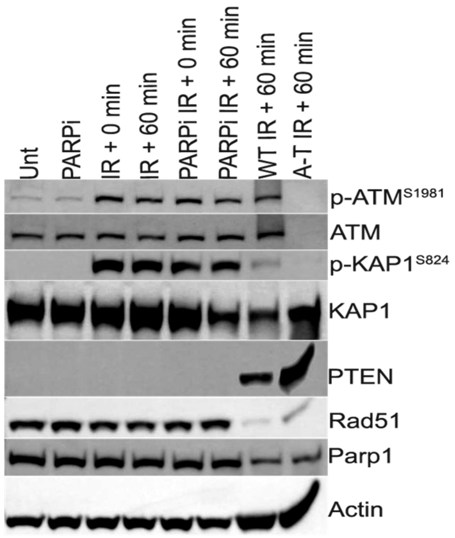

Figure S2. RAD51 is expressed in the PTEN-deficient Burkitt lymphoma cell line, BJAB. Irradiation of these cells (10 Gy) irrespective of PARP inhibition induces normal DNA damage responses, including ATM-dependent KAP-1 phosphorylation. Irradiation of immortalized lymphoblastoid cells (LCLs) derived from normal (WT) and ataxia–telangiectasia (A–T) patients serves to obtain ATM-proficient and -deficient control lines, respectively, to track ATM-mediated DNA damage signaling.

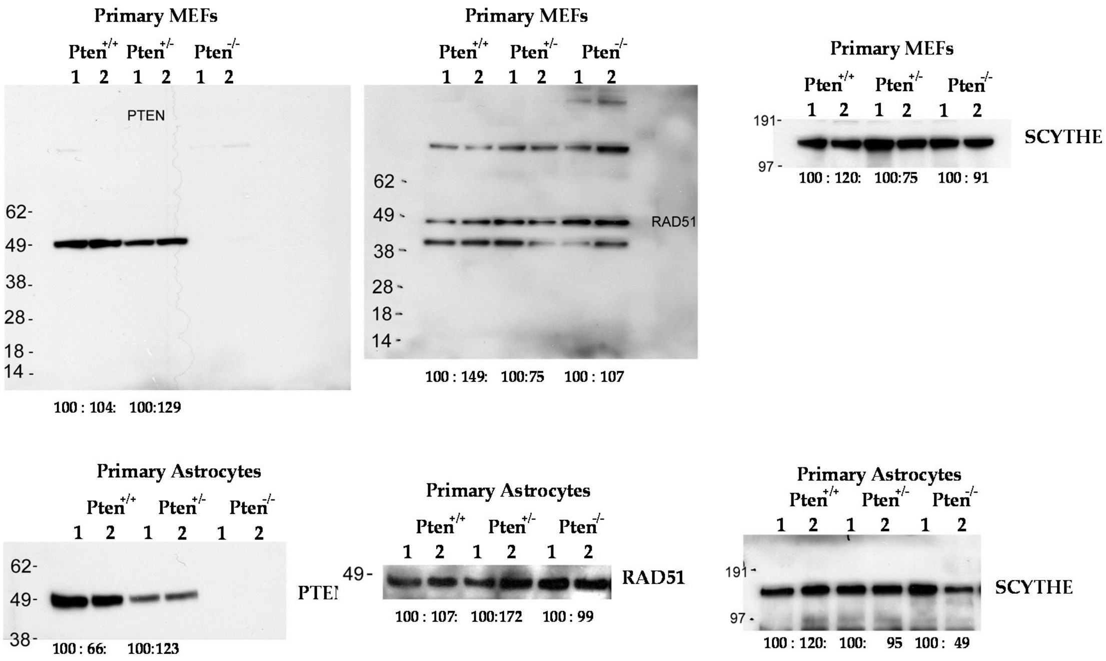

Uncropped Western Blot Figures for Figure 1.

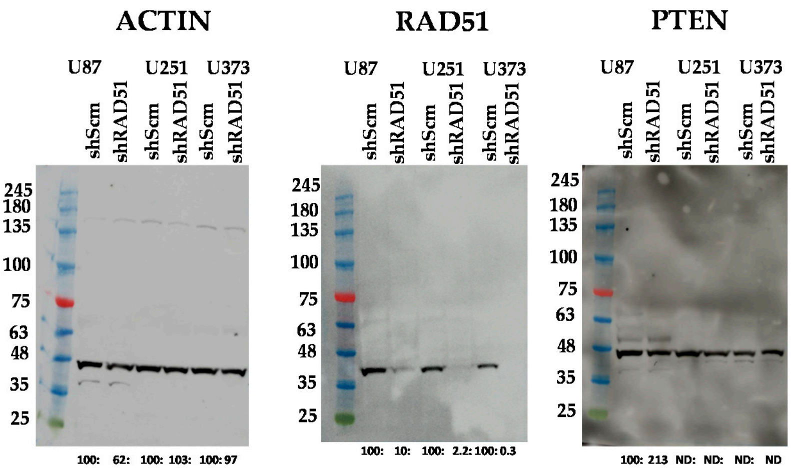

Uncropped Western Blot Figures for Figure 3.

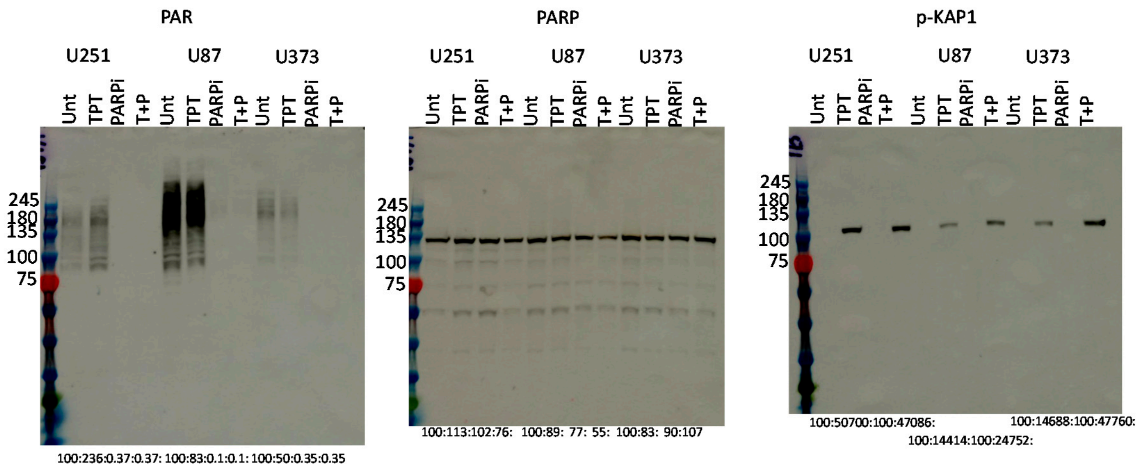

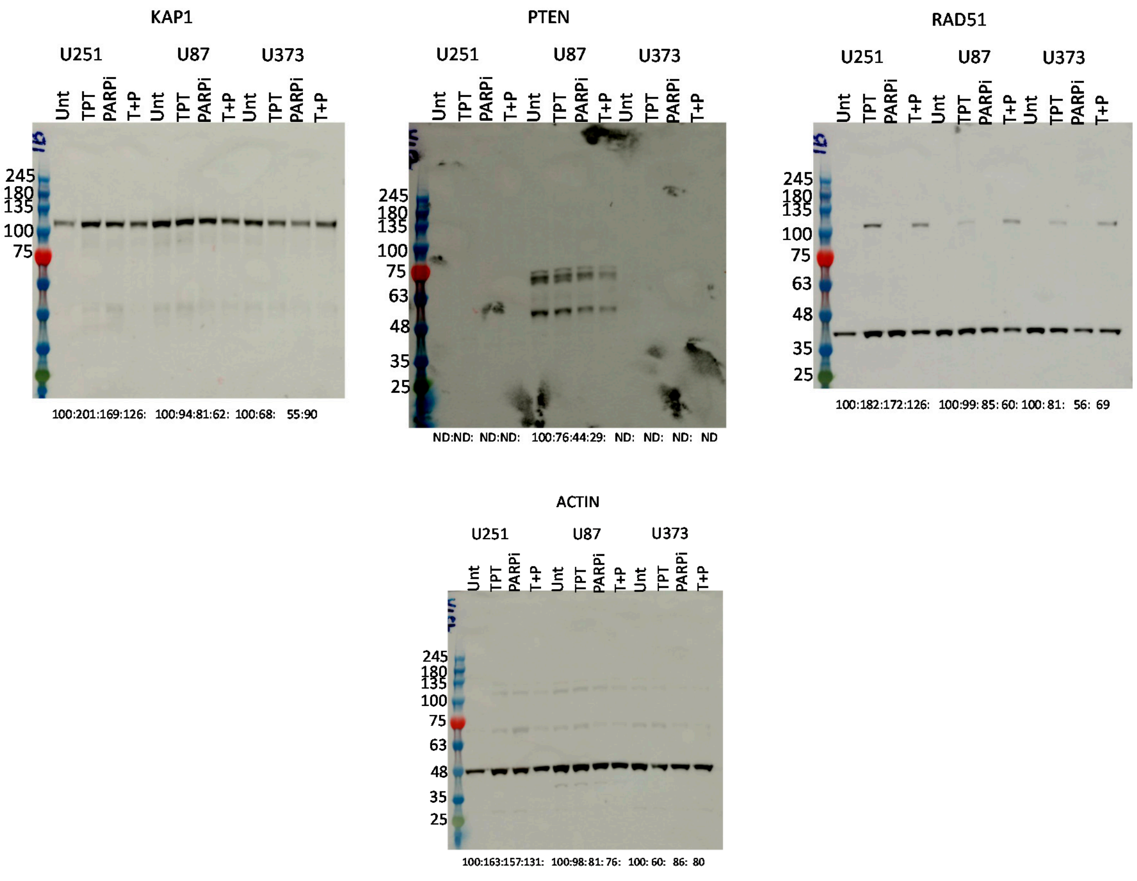

Uncropped Western Blot Figures for Figure 4.

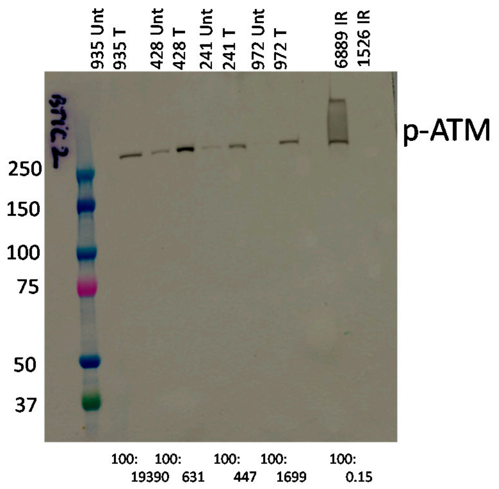
935 Unt
935 T
428 Unt
428 T
241 Unt
241 T
972 Unt
972 T
6889 IR
1526 IR
p-ATM
250
150
100
75
50
37
100: 100: 100: 100: 100:
19390 631 447 1699 0.15

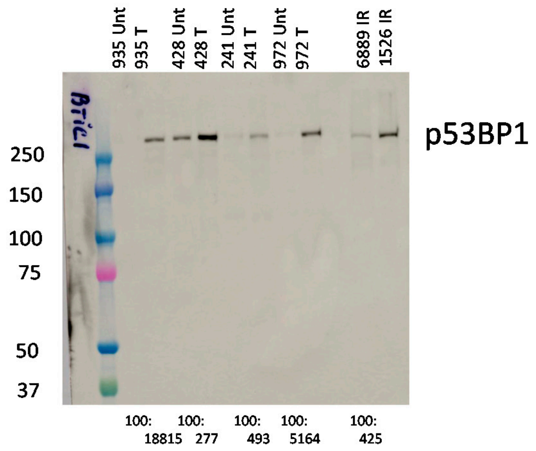
935 Unt
935 T
428 Unt
428 T
241 Unt
241 T
972 Unt
972 T
6889 IR
1526 IR
p53BP1
250
150
100
75
50
37
100: 100: 100: 100: 100:
18815 277 493 5164 425

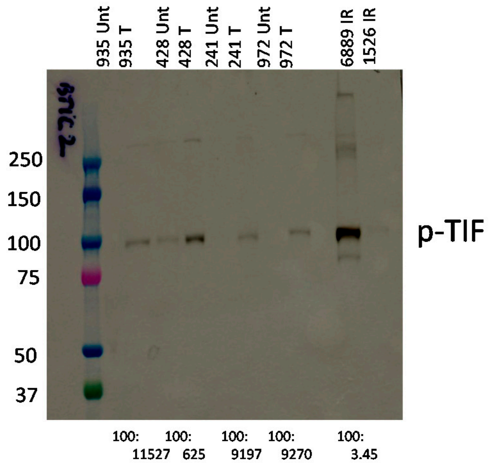
935 Unt
935 T
428 Unt
428 T
241 Unt
241 T
972 Unt
972 T
6889 IR
1526 IR
p-TIF
250
150
100
75
50
37
100: 100: 100: 100: 100:
11527 625 9197 9270 3.45

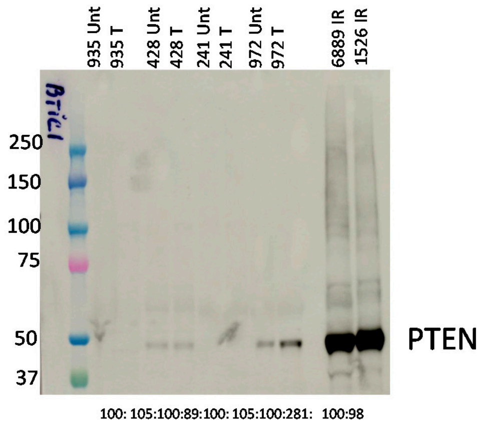
935 Unt
935 T
428 Unt
428 T
241 Unt
241 T
972 Unt
972 T
6889 IR
1526 IR
PTEN
250
150
100
75
50
37
100: 105:100:89:100: 105:100:281: 100:98

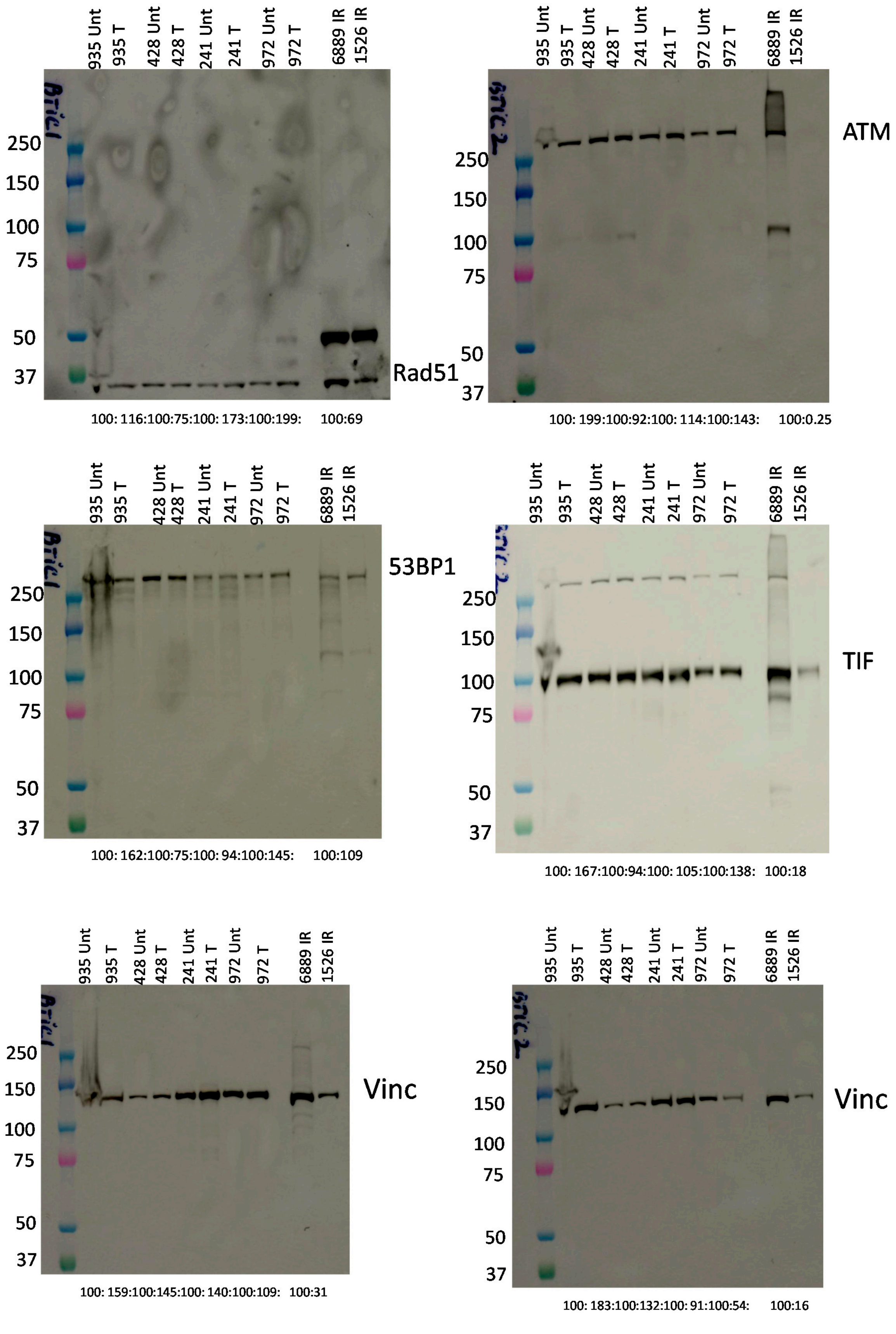

Uncropped Western Blot Figures for Figure 5.

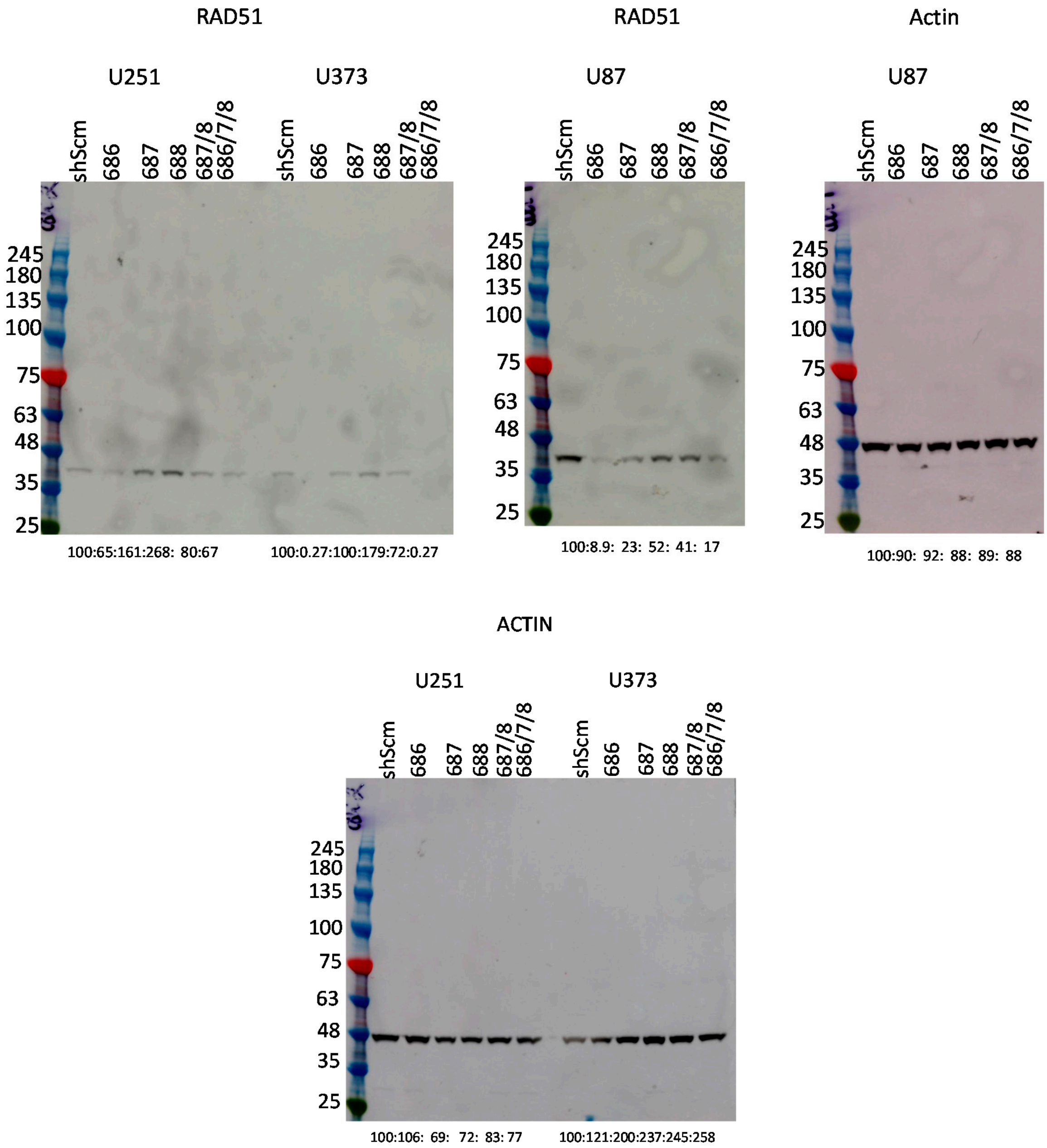

Uncropped Western Blot Figures for Figure S1.

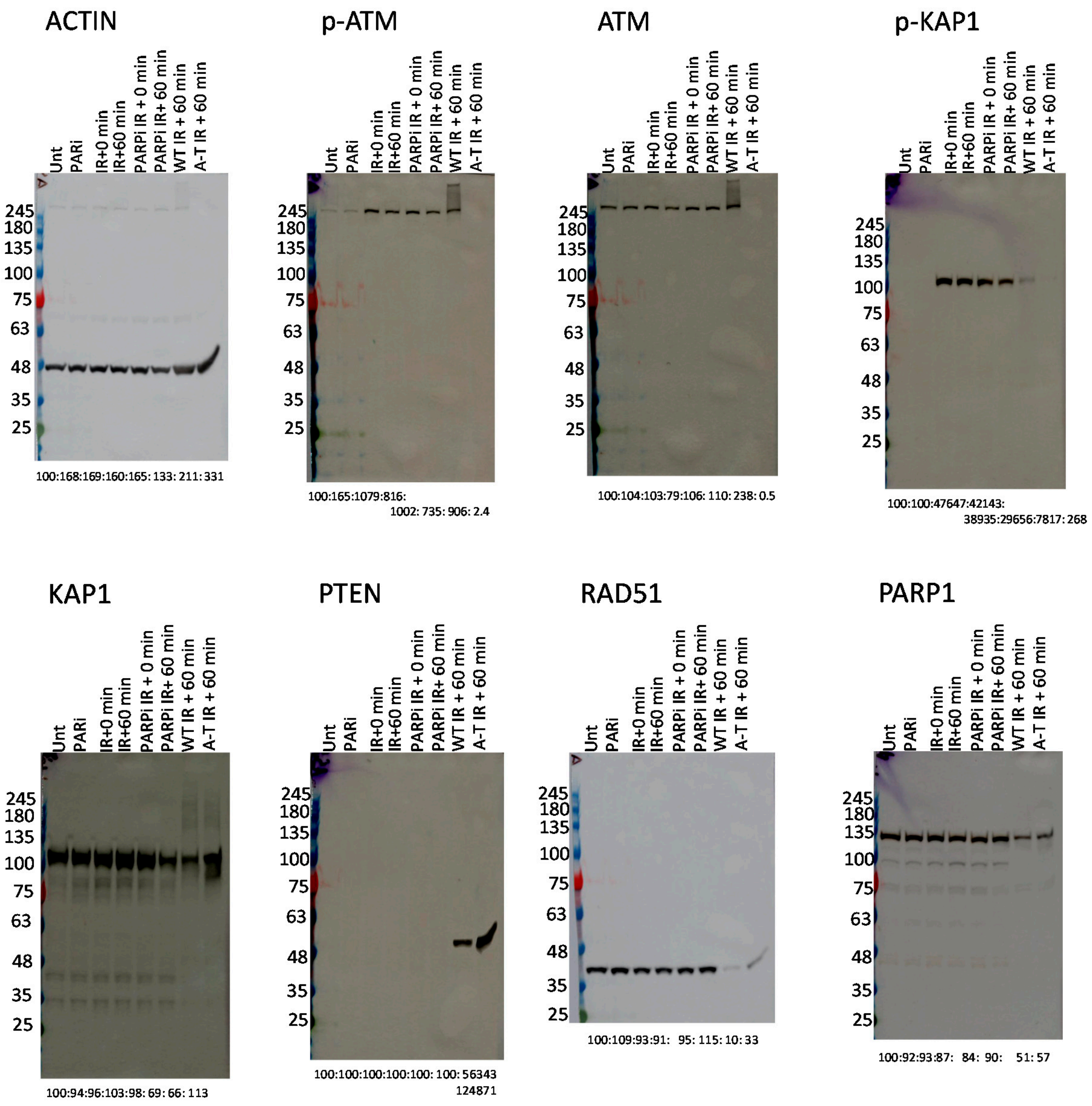

Uncropped Western Blot Figures for Figure S2.
